# Pharmacokinetics of Lusutrombopag, a Novel Thrombopoietin Receptor Agonist, in Rats by UPLC-MS/MS

**DOI:** 10.1155/2020/7290470

**Published:** 2020-05-30

**Authors:** Bo Wang, Feifei Chen, Quan Zhou, Yunfang Zhou, Deru Meng, Peiwu Geng, Ailian Hua, Weiping Ji, Changxiong Wang, Shuanghu Wang, Liming Hu

**Affiliations:** ^1^Department of Orthopaedics, The Sixth Affiliated Hospital of Wenzhou Medical University, The People's Hospital of Lishui, Lishui 323000, Zhejiang, China; ^2^The Laboratory of Clinical Pharmacy, The Sixth Affiliated Hospital of Wenzhou Medical University, The People's Hospital of Lishui, Lishui 323000, Zhejiang, China; ^3^Department of Pharmacy, The First People's Hospital of Wenling, Wenling 317500, Zhejiang, China

## Abstract

Lusutrombopag is a second oral thrombopoietin (TPO) receptor agonist that selectively acts on human TPO receptors. In the study, UPLC-MS/MS was used to establish a selective and sensitive method to determine lusutrombopag with poziotinib as IS (internal standard) in rat plasma. Samples were prepared by precipitating protein with acetonitrile as a precipitant. Separation of lusutrombopag and poziotinib was performed on a CORTECS UPLC C18 column (2.1 ∗ 50 mm, 1.6 *μ*m). The mobile phase (acetonitrile and water containing 0.1% formic acid) with gradient elution was set at a flow rate of 0.4 ml/min. The mass spectrometric measurement was conducted under positive ion mode using multiple reaction monitoring (MRM) of *m*/*z* 592.97 ⟶ 491.02 for lusutrombopag and *m*/*z* for poziotinib (IS) 492.06 ⟶ 354.55. The linear calibration curve of the concentration range was 2–2000 ng/ml for lusutrombopag, with a lower limit of quantification (LLOQ) of 2 ng/ml. RSD of interday and intraday precision were both no more than 9.66% with the accuracy ranging from 105.82% to 108.27%. The extraction recovery of lusutrombopag was between 82.15% and 90.34%. The developed and validated method was perfectly used in the pharmacokinetic study of lusutrombopag after oral administration in rats.

## 1. Introduction

Chronic liver disease (CLD) usually includes drug-induced liver disease, alcoholic liver disease, nonalcoholic fatty liver disease, viral hepatitis, liver cirrhosis, and liver cancer [1]. The incidence of CLD is increasing year by year in China and all over the world. CLD can participate in a system that alters the body's normal hemostasis and thrombosis [2]. The most common symptom is thrombocytopenia, with up to 76% of patients reporting lower than normal values [3, 4]. It is usually diagnosed or treated by invasive surgery, but the thrombocytopenia of the disease could significantly increase the risk of bleeding, which hinders the invasive treatment [5, 6]. Therefore, efforts are needed to control the occurrence of thrombocytopenia. Thrombopoietin (TPO) synthesized in the human liver can reduce its occurrence. TPO is a hormone used to increase platelet count, mainly regulating megakaryocytes and producing platelets [7]. Lusutrombopag is a second oral TPO receptor agonist that selectively acts on human TPO receptors and activates signaling pathways to produce megakaryocytes through a series of proliferation and differentiation of cells to increase platelet counts [8]. Lusutrombopag, an FDA-approved drug for the treatment of chronic liver disease, was approved for use in invasive surgery in Japan in September 2015 [9]. Therefore, further pharmacokinetic and pharmacological studies of lusutrombopag are feasible, and the methodological verification is first required.

Previous clinical study is to increase the safety of surgery by increasing the platelet count of lusutrombopag prior to invasive surgery [10]. To date, no literature has validated methodological studies on lusutrombopag to further explore the mechanism of action of drugs. In this study, the developed and validated method was perfectly used to quantify lusutrombopag in rat plasma and in the pharmacokinetic study of lusutrombopag after oral administration in rats by UPLC-MS/MS.

## 2. Materials and Methods

### 2.1. Chemicals and Reagents

Lusutrombopag and poziotinib (IS) with a purity of not less than 98% were both purchased from Beijing Sunflower and Technology Development Co., Ltd. (Beijing, China). Analytical grade formic acid was provided by Sigma-Aldrich (St. Louis, MO, USA). Chromatography grade acetonitrile and methanol were provided by Fisher Scientific Co. (Fair Lawn, New Jersey, USA). All other chemicals and reagents were of analytical grade.

### 2.2. Instruments and UPLC-MS/MS Conditions

Samples were analyzed by a UPLC-MS/MS system equipped with a triple quadrupole mass spectrometer (Waters Corp., Milford, MA, USA). Separation of lusutrombopag and poziotinib was performed using a CORTECS UPLC C18 column (2.1 × 50 mm, 1.6 *μ*m) maintained at 37°C. Acetonitrile (*A*) and water (0.1% formic acid, *B*) make up the mobile phase with gradient elution: 0.0–0.5 min (20% *A*), 0.5–1 min (rapidly rising from 20% *A* to 95% *A*), 1-2 min (maintained at 95% *A*), and 2–2.6 min (reduced to 20% *A*). The flow rate was set as 0.4 ml/min. All the compounds were analyzed within 3 min and the SM-FTN was washed after each injection with methanol-water solution.

**Figure 1:**
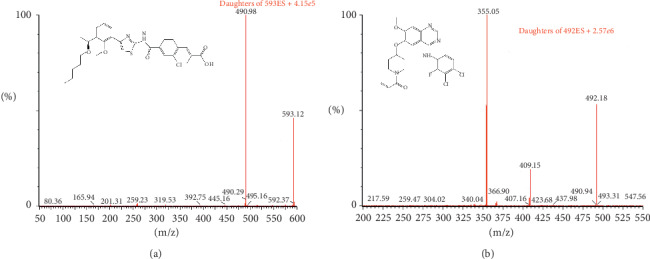
Mass spectra of lusutrombopag (a) and poziotinib (IS) (b).

The mass spectrometer was used to measure by a triple quadrupole mass analyzer with better specificity, selectivity, and sensitivity and an electrospray ionization mode that causes ions that pass through to repel ions from the droplets. The optimal MS parameters were defined as follows: source temperature 150°C, desolvation temperature 500°C, capillary voltage and cone voltage 4 kV and 30 V, collision energy 20 V and 28 V for lusutrombopag and IS, respectively. The fragment ions *m*/*z* 592.97 ⟶ 491.02 for lusutrombopag and *m*/*z* 492.06 ⟶ 354.55 for poziotinib were used for quantitative analysis. Qualitative analysis fragment ion for lusutrombopag was *m*/*z* 592.97 ⟶ 258.96 (Figure 1). All sample data were acquired and the instrument was controlled by MassLynx 4.1 software (Waters Corp.).

### 2.3. Preparation of Standard and Quality Control (QC) Samples

The stock solution of lusutrombopag (0.5 mg/mL) was manufactured in methanol and water, so as poziotinib (0.5 mg/mL, IS). A working standard solution of lusutrombopag and IS was prepared by adding the stock solution and methanol. The lusutrombopag solution was added to the blank rat plasma, and the standard curve was constructed at 2–2000 ng/mL: 2, 5, 10, 50, 100, 250, 500, 1000, and 2000 ng/mL. In a similar manner, three plasma concentrations (4, 40, and 1600 ng/ml) of lusutrombopag quality control (QC) samples were prepared separately, and their storage environment was −20°C.

### 2.4. Sample Preparation

Plasma samples were allowed to reach room temperature and vortexed before LC-MS analysis. 20 *μ*L of an equal amount of IS solution (0.5 *μ*g/mL) per sample, spiked with 50 *μ*L of the obtained plasma sample, and 100 *μ*L acetonitrile were added to a 1.5 mL centrifuge tube and then vortexed for 1 min. The mixtures were centrifuged for 10 minutes at a speed of 13,000 rpm, followed by injecting 2 *μ*L supernatant into a UPLC-MS/MS system.

### 2.5. Method Validation

Methodology validation requires verification of linearity, accuracy, precision, recovery, and stability which were tested strictly, referring to the appropriate literature guidelines (FDA [11] and EMA [12]), in order to comprehensively and deeply verify bioanalytical methods [13–16]. The validation run of one set of standard and five QC quality control samples was performed within three consecutive days. The rat blank plasma was mixed with the prepared lusutrombopag in three different concentrations (4, 400, and 1600 ng/mL) of working solution to prepare three (HQC, MQC, and LQC) samples of lusutrombopag.

#### 2.5.1. Specificity

The specificity was verified by qualitatively analyzing rat blank plasma, adding blank plasma of lusutrombopag and IS and rat plasma samples of oral lusutrombopag for 4 h, thereby comparing the integrity and endogenousity of the UPLC-MS/MS chromatogram of the blank and the verified samples, while some endogenous material interferences were also avoided.

#### 2.5.2. Linearity and Lower Limit of Quantification (LLOQ)

Calibration curves were drawn by selecting standard samples on three separate days. Regression analysis was performed on the ratio of the response of lusutrombopag to IS to the drug concentration by weighted least squares method to draw a standard curve. LLOQ is the lowest point in the working curve, with a signal/noise ratio ≥10 and the precision (RSD) and the accuracy (R.E.) ≤20%.

#### 2.5.3. Accuracy and Precision

QC samples were selected at three concentration levels (4, 400, and 1600 ng/mL) for three consecutive days to verify accuracy and precision. The precision, including intraday and interday precision, was obtained by repeating the determination of six QC samples in the same day or 6 consecutive days. Accuracy was the closeness between the average of the measured sample and the primary concentration. Precision and accuracy were required to be within ±15%.

#### 2.5.4. Recovery and Matrix Effect

The recovery of lusutrombopag was calculated by the extracted QC samples with standard control solution in the blank plasma (*n* = 6). The recovery rate of IS was also measured by the similar way. The matrix effect was evaluated by the ratio of the peak area of lusutrombopag extracted from blank plasmas with those dissolved in pure standard solution at three concentration levels (4, 400, and 1600 ng/mL) (*n* = 6).

#### 2.5.5. Stability

The stability of QC samples for lusutrombopag were studied under different possible conditions, which included stability of three cycles of freeze/thaw (−20°C and room temperature), stability of the analytes at 4 °C for 6h and room temperature for 24 h, and stability of plasma samples stored at -20°C for 14 days (*n* = 6). These results were compared with the measured values of the newly prepared QC samples under the same conditions. IS was also processed in a similar way to estimate the stability.

### 2.6. Application to Pharmacokinetic Studies

All twelve male Sprague Dawley rats (220 ± 20 g) were supplied by the Animal experiment center of Wenzhou Medical University. Rats lived in an environment where temperature and humidity were relatively suitable and were free to obtain food and water until 18 hours before the start of the experiment. All experimental procedures and protocols were reviewed and adopted by the Animal Care and Use Committee of Wenzhou Medical University (no. wydw2017-0010). Rats fasted 12 hours before the drug was administrated, but there was free access to water. The oral dose of lusutrombopag (dissolved in CMC-Na solution) was 10 mg/kg each time. After oral administration of lusutrombopag 0.083, 0.25, 0.5, 1, 1.5, 2, 4, 6, 8, 12, 24, 36, and 60 h, blood samples (0.3 mL) were taken from the tail vein of the rats into a 1.5 mL centrifuge tube (heparin was added to the tube to prevent blood clotting). The samples were immediately centrifuged at 4000 g for 10 min to obtain the supernatant, which was taken and stored at −20°C until analysis. The plasma was kept at −20°C until analyses. Pharmacokinetic parameters of lusutrombopag were analyzed as follow: maximum plasma concentration (*C*_max_), area under the plasma concentration–time curve (AUC), plasma clearance (CL), and half-life (*t*_1/2_) were calculated using DAS software (Drug and Statistics, version 3.2.8, The People's Hospital of Lishui, China) and by a noncompartmental model.

## 3. Results and Discussion

### 3.1. Method Development

Mass spectrometers equipped with an electrospray ionization (ESI) interface produce higher sensitivity in mass spectrometry; lusutrombopag and IS acquired the protonated precursor molecule [M + H]^+^. Our results show that, for lusutrombopag, the main ions detected were 592.97 ⟶ 491.02 for lusutrombopag and m/*z* 492.06 ⟶ 354.55 for poziotinib (IS). The choice of different mobile phases and columns played a crucial role in producing satisfactory chromatographic results. Elution with acetonitrile andwater (containing 0.1% formic acid) as the mobile phase presented a more satisfactory peak shape and suitable retention time. Each run time was 3 min, and the retention times of lusutrombopag and IS were 1.75 min and 1.25 min. The CORTECS UPLC C18 column (2.1 ∗ 50 mm, 1.6 *μ*m) was found to give more satisfactory chromatographic results than the ACQUITY UPLC HSS T3 column (2.1 ∗ 100 mm, 1.8 *μ*m). A key step before performing LC-MS analysis is to ensure removal of other potential interferences, including proteins and biological samples [17]. In this study, protein precipitation with acetonitrile provided better matrix effects and higher recovery than liquid-liquid extraction.

### 3.2. Specificity

**Figure 2:**
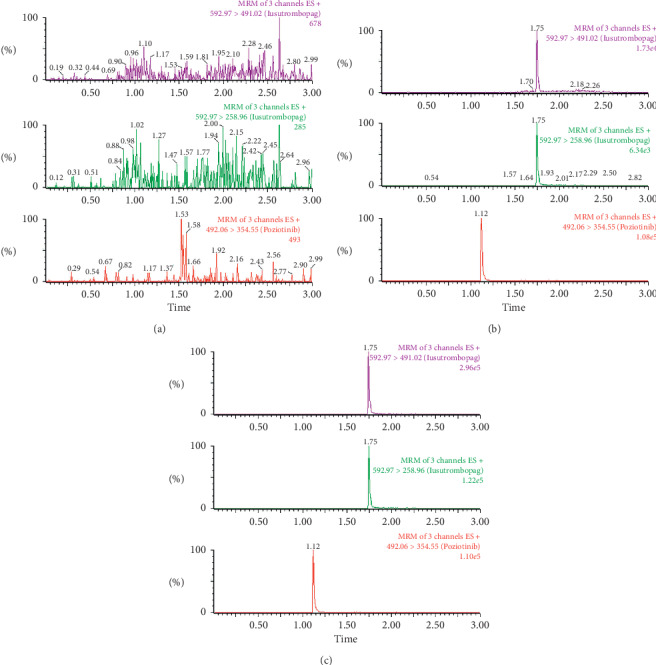
Representative UPLC-MS/MS chromatograms of lusutrombopag and poziotinib (IS). (a) Blank plasma; (b) blank plasma spiked with lusutrombopag (2 ng/mL)and IS (50 ng/mL); (c) a rat plasma sample taken 4 hours after the oral dose of 10 mg/kg lusutrombopag.

The chromatograms of blank plasma, a plasma sample spiked with lusutrombopag and IS, and a sample obtained at 4 h after oral administration of 10 mg/kg lusutrombopag are shown in Figure 2. There were no interfering endogenous substances observed between the peaks of lusutrombopag or IS.

### 3.3. Linearity and the LLOQ

The standard curve of lusutrombopag rat plasma was linear from 2 to 2000 ng/mL. A typical regression equation is *y* = 0.0154811*x* + 0.0994656, including *y* for the ratio of the peak area of lusutrombopag to IS and *x* for the drug concentration in the standard sample. The LLOQ of lusutrombopag was measured at 2 ng/mL. Calibration curves of lusutrombopag in plasma, assessed by performing back-calculated concentrations, showed <15% deviation from nominal values at all concentrations, and the deviation was <20% for the LLOQ.

### 3.4. Precision, Accuracy, Recovery, and Matrix Effect

Three different concentrations of QC samples were usually selected for precision and accuracy and within three different days. As shown in Table 1, the interday precision was 9.66% or lower and the intraday precision is 7.99% or lower at each QC levels. The accuracy of the method was between 105.82% and 108.27%. These results were consistent with the accuracy and precision (RSD) within ±15% acceptance criteria, indicating that these values are within the measurement range with satisfactory accuracy and precision. The average recovery of Lusutrombopag was 86.5%. The matrix effect of lusutrombopag ranging from 82.84 to 92.47 showed that plasma matrix did not significantly affect the results.

### 3.5. Stability

Table 2 shows that lusutrombopag at different concentrations (4, 400, and 1600 ng/mL) was stable in rat plasma at the following conditions: 24 hours at room temperature, 6 hours at 4°C, 3 repeated freeze-thaw cycles (stored at −20°C and thawed at room temperature) and store at −20°C for 14 days, with all the average concentration limits of the measured values within ±15%. Thus, all the analytes were stably stored under the conditions mentioned above.

### 3.6. Pharmacokinetic Study

**Figure 3:**
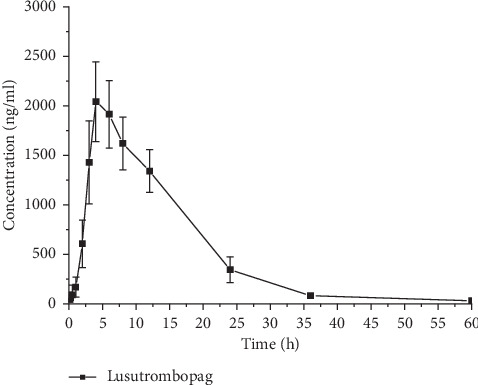
Mean plasma concentration-time curve of lusutrombopag in rats (*n* = 6, mean ± SD).

The method for the determination of rat plasma and pharmacokinetic study of lusutrombopag was successfully applied in rats, with the drug-time curve shown in Figure 3. The pharmacokinetic parameters of oral administration (10 mg/kg) were calculated in a noncompartmental model shown in Table 3. The data showed that *C*_max_ was 2187.22 ± 279.56 ng/mL after oral sarecycline. After 3 hours, the blood drug concentration began to decrease. The drop in drug concentration might lie in the distribution from plasma to other organs and tissues.

## 4. Conclusions

UPLC-MS/MS was able to rapidly and accurately determine lusutrombopag in rat plasma with a LLOQ concentration of 2 ng/mL. The pharmacokinetic study of lusutrombopag after oral administration has been demonstrated to be described by UPLC-MS/MS. As far as one knows, it is the first description of the pharmacokinetic profile of lusutrombopag in rat plasma. The validated method was perfectly used in the pharmacokinetic study of lusutrombopag after oral administration in rats.

## Figures and Tables

**Table 1:** Precision, accuracy, and recovery for lusutrombopag of QC samples in rat plasma (*n* = 6).

Concentration (ng/mL)	RSD		Accuracy		Recovery (%)	Matrix effect (%)
	Intraday (%)	Interday (%)	Intraday (%)	Interday (%)		
4.00	7.12	9.66	108.13	105.82	82.15	82.84
400.00	7.99	2.40	109.51	108.27	90.34	84.80
1600.00	1.86	1.40	107.20	106.58	86.88	92.47

**Table 2:** Summary of stability of lusutrombopag and IS under various storage conditions (*n* = 6).

Condition	Concentration (ng/mL)		RSD (%)	Accuracy (%)
	Added	Measured		
Room temperature, 24 h	4	4.04 ± 0.27	6.63	101.01
	400	454.94 ± 25.56	5.62	113.74
	1600	1705.61 ± 84.80	4.97	106.60
Freeze-thaw	4	4.53 ± 0.18	3.98	113.36
	400	421.27 ± 19.55	4.64	105.32
	1600	1702.84 ± 45.14	2.65	106.43
4°C, 6 h	4	4.17 ± 0.20	4.75	104.33
	400	440.99 ± 36.80	8.34	110.25
	1600	1626.84 ± 57.93	3.56	101.68
−20°C, 14 d	4	4.25 ± 0.29	6.77	106.19
	400	443.77 ± 53.63	12.09	110.94
	1600	1660.99 ± 33.63	2.02	103.82

**Table 3:** Primary pharmacokinetic parameters after oral administration of lusutrombopag in rats (*n* = 6).

Parameter	Unit	Oral administration (*n* = 6)
AUC_(0–*t*)_	*μ*g/L·h	30739.23 ± 6067.41
AUC_(0–∞)_	*μ*g/L·h	30864.05 ± 6204.74
MRT_(0–*t*)_	h	12.30 ± 0.88
MRT_(0–∞)_	h	12.50 ± 0.97
*t *_1/2*z*_	h	6.57 ± 1.37
*T *_max_	h	5.17 ± 1.84
Vz/*F*	L/kg	3.08 ± 0.30
CLz/*F*	L/h/kg	0.34 ± 0.07
*C *_max_	*μ*g/L	2187.22 ± 279.56

## Data Availability

The data used to support the findings of this study are available from the corresponding author upon request.
